# Let's keep in touch: How membrane contact sites drive immune responses

**DOI:** 10.1093/plcell/koaf229

**Published:** 2025-09-27

**Authors:** Sonhita Chakraborty

**Affiliations:** Assistant Features Editor, The Plant Cell, American Society of Plant Biologists

Inside every cell, a pulsating network of molecular messengers orchestrates life's most critical conversations. While vesicular trafficking facilitates long-distance communication, short-range intracellular communication is best accomplished between organelles that are in close proximity and tethered together. This allows them to be in close contact while maintaining their respective membranes. Membrane contact sites (MCS) mediate the exchange of traffic cargo and materials between organelles and have been implicated in playing important roles during autophagy, calcium signaling, organelle biogenesis, and lipid metabolism (Voeltz et al. 2024). In plants, MCS have also been shown to be important in stress responses (Wang et al. 2023). However, we do not fully understand how organelles and MCS engage dynamically during stress responses, especially how they coordinate their positioning and interactions to effectively mount immunity signaling in response to invading pathogens.

When pathogens like oomycetes and fungi infect their plant hosts, they develop penetrating haustoria that cause the host membrane to bend. Haustoria become enveloped by a host-derived extrahaustorial membrane (EHM), which differs from the plasma membrane in protein and lipid composition (Bozkurt and Kamoun 2020). At the EHM, part of the host plasma membrane that encases haustoria, pathogens derive nutrients from the host and release effectors to suppress the plant's immune responses (Petre et al. 2021; Yuen et al. 2023). In turn, plants polarize their defenses toward the EHM, reinforcing it through callose deposition and other focal immune responses (King et al. 2024). Organelles like mitochondria and chloroplasts have been shown to accumulate at EHMs (Fuchs et al. 2016; Savage et al. 2021). Notably, chloroplasts form tubular extensions, termed stromules, that associate with the EHM surrounding the haustoria of the oomycete *Phytophthora infestans* (*P. infestans*) (Savage et al. 2021). However, the mechanisms guiding chloroplast positioning and their contribution to immunity have not been fully uncovered. In new work, **Enoch Lok Him Yuen and colleagues (Yuen et al. 2025)** explore chloroplast positioning in response to *P. infestans* infection and the combined role of 2 proteins in anchoring chloroplasts at MCS.

The chloroplast outer-envelope actin polymerization factor CHLOROPLAST UNUSUAL POSITIONING 1 (CHUP1) has been implicated in anchoring chloroplasts and mediating effector-triggered immunity (Nedo et al. 2024). Now, Yuen et al. found that either silencing or knocking out CHUP1 increased plant susceptibility to *P. infestans*. However, chloroplast positioning around haustorium did not differ between *chup1* and control plants, as quantified by the number of chloroplast-haustoria associations. These findings suggest that the enhanced disease susceptibility associated with the loss of CHUP1 is not caused by impaired chloroplast movement. By tracking transiently expressed CHUP1-GFP in the solanaceous model plant *Nicotiana benthamiana*, Yuen et al. (2025) observed that CHUP1 accumulates at the chloroplast-plasma membrane and chloroplast-EHM MCS. To further decipher CHUP1's role in mounting focal immunity against *P. infestans*, the authors investigated how core immune pathways, such as those associated with PAMP- or effector-triggered immunity, were altered in *chup1*. Although the activation of core immune pathways was not affected, callose deposition, typically associated with focal immunity, was significantly reduced in *chup1*. This suggested that CHUP1 acts downstream of the core immunity activation pathways and that its role in tethering chloroplasts to EHMs is important in mediating the focal immune responses of callose deposition.

Next, the authors turned their attention to KINESIN-LIKE PROTEIN FOR ACTIN-BASED CHLOROPLAST MOVEMENT (KAC), which was previously implicated in chloroplast movement and membrane association (Suetsugu et al. 2012). Silencing KAC not only enhanced susceptibility to *P. infestans* but also impaired callose deposition in response to the pathogen. Having identified CHUP1 and KAC as proteins with seemingly similar roles in mediating focal immunity, Yuen et al. (2025) studied the localization patterns of CHUP1 and KAC1. Transient coexpression of CHUP1–GFP and KAC1–RFP, together with in planta co-immunoprecipitation assays, confirmed their co-localization at chloroplast MCS with both the EHM and the plasma membrane, as well as their physical interaction. These findings suggest that CHUP1 and KAC1 might act together in coordinating chloroplast docking at MCS.

By revealing that CHUP1 and KAC cooperate to tether and dock chloroplasts at MCS, thereby facilitating focal immunity during *P. infestans* infection (Fig.), this paper deepens our understanding of the critical role MCS play in plant-pathogen interactions. While the mechanisms by which these proteins form MCS, tether chloroplasts to the EHM, or subsequently mediate callose deposition are still unknown, the work by Yuen and colleagues (2025) opens exciting avenues for understanding contact site dynamics and developing biotechnological methods of improving crop resilience to microbial pathogens.

**Figure.**
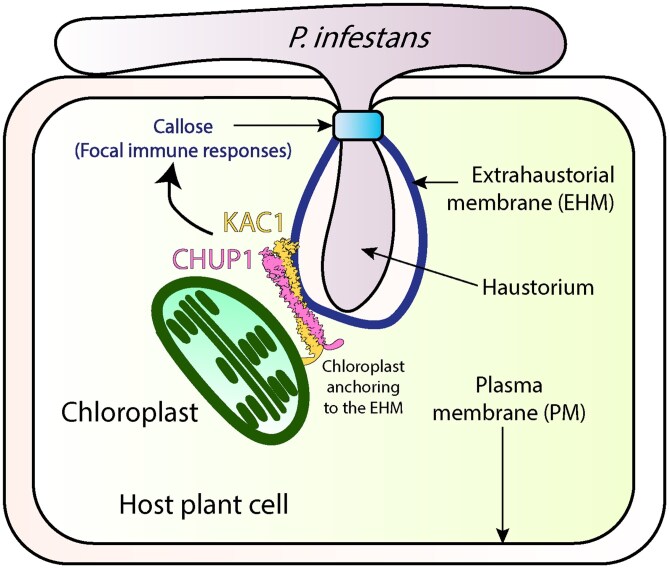
Overview of how CHUP1 and KAC1 help anchor chloroplasts to the EHM during plant immune response. As the haustorium of *P. infestans* penetrates its host, it stimulates the formation of a host-derived EHM at the plant-pathogen interface. In response, the chloroplast movement protein CHUP1 and the microtubule motor kinesin-like protein KAC1 anchor chloroplasts to the EHM and initiate the formation of MCS. These MCS are thought to play an important role in mediating plant focal immunity. Reprinted from Yuen et al. (2025), Figure 6.

## Recent related articles in *The Plant Cell*

- Ostermeier et al. (2024) comprehensively reviewed how thylakoid membrane architectures are formed and maintained and how they have evolved in phototrophic organisms.
- Zhuang et al. (2024) provided a historical overview of key discoveries, tools, and models that have shaped our understanding of organelle structure, dynamics, and interactions within the plant endomembrane system.
- Xue et al. (2025) demonstrated how powdery mildew infection in *Arabidopsis* supports fungal spore production by remodeling host leaf lipid metabolism and promoting localized triacylglycerol (TAG) accumulation at the expense of thylakoid membrane lipids.
- Kong et al. (2024) uncovered how the Arabidopsis CHUP1 functions as a plant-specific actin-binding protein that regulates chloroplast positioning and movement through blue light– and phototropin-dependent mechanisms.

## Data Availability

No new data were generated or analysed in support of this research.

## Dive Curated Terms

The following phenotypic, genotypic, and functional terms are of significance to the work described in this paper:

CHUP1 Gramene: AT3G25690
CHUP1 Araport: AT3G25690
